# Alignment of Supermarket Own Brand Foods’ Front-of-Pack Nutrition Labelling with Measures of Nutritional Quality: An Australian Perspective

**DOI:** 10.3390/nu10101465

**Published:** 2018-10-09

**Authors:** Claire Elizabeth Pulker, Georgina S. A. Trapp, Jane Anne Scott, Christina Mary Pollard

**Affiliations:** 1 School of Public Health, Curtin University, Kent Street, GPO Box U1987, Perth 6845, Western Australia, Australia; jane.scott@curtin.edu.au (J.A.S.); c.pollard@curtin.edu.au (C.M.P.); 2 Telethon Kids Institute, The University of Western Australia, P.O. Box 855, West Perth 6872, Western Australia, Australia; gina.trapp@telethonkids.org.au; 3 School of Population and Global Health, The University of Western Australia, 35 Stirling Highway, Crawley 6009, Western Australia, Australia; 4 East Metropolitan Health Service, Kirkman House, 20 Murray Street, East Perth 6004, Western Australia, Australia

**Keywords:** Health Star Rating, Daily Intake Guide, front-of-pack label, supermarket, supermarket own brand, private label, nutrition

## Abstract

Two voluntary front-of-pack nutrition labels (FOPNL) are present in Australia: the government-led Health Star Ratings (HSR) and food industry-led Daily Intake Guide (DIG). Australia’s two largest supermarkets are key supporters of HSR, pledging uptake on all supermarket own brand foods (SOBF). This study aimed to examine prevalence of FOPNL on SOBF, and alignment with patterns of nutritional quality. Photographic audits of all SOBF present in three large supermarkets were conducted in Perth, Western Australia, in 2017. Foods were classified as nutritious or nutrient-poor based on the Australian Guide to Healthy Eating (AGTHE), NOVA level of food processing, and HSR score. Most (81.5%) SOBF featured FOPNL, with only 55.1% displaying HSR. HSR was present on 69.2% of Coles, 54.0% of Woolworths, and none of IGA SOBF. Half (51.3%) of SOBF were classified as nutritious using the AGTHE, but using NOVA, 56.9% were ultra-processed foods. Nutrient-poor and ultra-processed SOBF were more likely than nutritious foods to include HSR, yet many of these foods achieved HSR scores of 2.5 stars or above, implying they were a healthy choice. Supermarkets have a powerful position in the Australian food system, and they could do more to support healthy food selection through responsible FOPNL.

## 1. Introduction

Front-of-pack nutrition labels (FOPNL) have the potential to provide consumers with a convenient guide to healthy food selection [1]. It is a highly contested area of food labelling [2], and a variety of scoring systems and visual devices exist. They include initiatives from the food industry (e.g., the international Choices Program [3]), government agencies (e.g., the UK traffic lights [4]), and some supermarket scoring systems that are applied to shelf-edge labels of all foods (e.g., Guiding Stars [5]). These FOPNL have been described using a continuum with reductive (i.e., facts only, with no evaluation or recommendation) at one end, and evaluative (i.e., presence of the device indicates compliance with predefined criteria) at the other, with hybrid or interpretive (i.e., a combination of facts and symbols) in the middle [6]. They have also been categorised as nutrient-specific systems (i.e., display the amount per serving of selected nutrients), summary indicator systems (i.e., a single symbol, icon, or score is used to summarise the nutrient content), and food group information systems (i.e., symbols are used to indicate presence of a specific food group) [1]. The policy objectives of each initiative can differ, with some labelling systems which have been designed according to best practice for effective product labels more likely to lead to changes in consumer purchasing behaviour than others [1,7]. The American Institute of Medicine recommends use of a single standardised FOPNL, which appears on all products in settings such as supermarkets, is promoted to consumers, and encourages reformulation of processed foods [8]. In addition, FOPNL that are led or endorsed by governments and international health agencies are generally regarded as the most credible [9].

### 1.1. FOPNL in Australia

FOPNL is voluntary in Australia, and there are two commonly applied labelling systems. The government-led Health Star Rating system (HSR) was designed to guide selection of healthier packaged foods, and uses an nutrient profiling algorithm to assign each product a score from ½ to 5 health stars, with 5 stars indicating the healthiest choice [10]. Launched in 2014, the original policy aim of the HSR was to guide consumers who have a wide range of literacy and numeracy skills to select healthier foods by enabling comparison between individual foods, and increasing awareness of the nutritional quality of foods, consistent with national dietary guidelines [11,12]. The objective of the HSR is summarised as: “*To provide convenient, relevant and readily understood nutrition information and/or guidance on food packs to assist consumers to make informed food purchases and healthier eating choices*” [13]. The food industry-led Daily Intake Guide (DIG), introduced in 2006 [14], aims to inform food selection by providing nutrition information for a serving of the product on the front-of-pack, along with contribution to the daily intake of an average adult [15] (Supplementary Figure S1). Comparison of the impact of the two Australian systems on consumer food choice concluded that interpretive labels (or summary indicator systems) such as the HSR can be more effective than reductive labels (or nutrient-specific systems) such as the DIG in guiding food selection [16,17]. However, a New Zealand randomised controlled trial which compared the effectiveness of traffic light labels, HSR and a control found the interpretive nutrition labels had no effect on food purchases [18]. The authors attributed this finding in part to low uptake of HSR on packaged foods in New Zealand and lack of awareness of the system [18]. To date, there is a lack of ‘real world’ evidence of the effectiveness of government-led FOPNL on consumer purchasing behaviour [7]. Therefore, the need to evaluate the impact of such labelling initiatives on public health continues.

Development of the HSR in Australia has not benefitted from the same levels of transparency given to development of FOPNL and nutrient profiling criteria in countries such as the UK [19,20,21] and France [22,23,24,25,26]. Although there has been considerable effort made by public health researchers to assess the potential impact of FOPNL on consumer purchasing behaviour since implementation of the HSR, there were no peer-reviewed studies that informed the development, validation, or implementation of the system [6]. This may have been because the HSR algorithm was not specifically designed from scratch to meet its policy aim. HSR system developers utilised the nutrient profiling criterion adopted by Food Standards Australia New Zealand (FSANZ) to determine whether foods were eligible to make health claims on packaging, which were in turn based on the UK nutrient profiling criterion used by Ofcom to determine whether foods could be advertised to children [27]. While the UK model is the most widely used and validated (for its purpose), adaptation from the original categorical scoring to continuous scoring for a mnemonic device on pack required technical decision-making, including setting HSR score cut-offs, that is currently unknown. In addition, a recent evaluation found that alignment between the two Australian nutrient profiling systems (i.e., HSR and FSANZ health claims) needed improving [28]. 

Given this lack of transparency, Australian researchers have sought to determine the ability of the HSR to assist consumers to select foods consistent with the recommendations of the Australian Dietary Guidelines [12]. To assist consumers to select the recommended nutritious five food group foods (a) the algorithm that underpins the HSR needs to correctly allocate scores that are consistent with national dietary guidelines; (b) the HSR should be widely applied to packaged foods; and (c) consumers who have a wide range of literacy and numeracy skills should understand how to use the HSR to guide selection of nutritious foods. The ability of the HSR to identify nutritious foods has been examined by studies seeking to determine whether total sugar should be substituted with added sugar in the HSR algorithm [29], and test the accuracy of HSR scores for dairy foods [30]. Researchers have attempted to measure uptake of the voluntary HSR on packaged foods [31], and examine the preference and ability of consumers to use HSR in several studies [17,32,33,34,35,36]. In addition, HSR may encourage reformulation of packaged food to improve the nutrient profile, which has been assessed in New Zealand [37] and Australia [38].

Two studies have specifically assessed congruence between HSR and the Australian Dietary Guidelines [12], with important differences in findings [39,40]. One concluded that the “*scope of genuine misalignment between the [Australian Dietary Guidelines] and HSR algorithm across the Australian food supply is very small*” [39] (p. 11), while the other concluded that “*the HSR system is undermining the [Australian Dietary Guideline] recommendations*”, as it did not consistently demarcate between nutritious and nutrient-poor foods [40] (p. 11). The difference in findings from these two studies can be explained by examining the methodologies employed, summarised in Table 1. Methodological decisions made about extracting or calculating the HSR score, rigour of food group classification, and allocating HSR cut-off points that are deemed appropriate to indicate nutritious and nutrient-poor foods, can influence study findings regardless of sample size. Questions about the ability of HSR to assist Australian consumers to select nutritious foods therefore remain.

### 1.2. FOPNL on Supermarket Own Brand Foods

Supermarkets have been identified as key supporters of FOPNL. In Australia, the two dominant supermarket chains who account for over 70% of Australian grocery sales [44] pledged to implement the HSR on all supermarket own brand foods (SOBF) [45], and therefore stop using the DIG [14]. SOBF (also known as private label, in-house brand, store brand, retailer brand, or home brand) are owned by retailers, wholesalers or distributors and sold privately in their own stores [46]. These products make a significant contribution to the global food supply and are predicted to grow until they dominate, led by the world’s largest supermarket chains [47]. In Australia, SOBF are predicted to reach 35 percent of grocery sales by 2020 [48]. Other packaged foods, or branded foods (also known as national brands, manufacturer brands, premium brands), are owned by food manufacturers [46]. Data from 2017 indicated two supermarket chains, Coles and Woolworths, and discount retailer Aldi contributed over half of the products adopting the HSR in Australia [31]. Similarly, a UK study found that almost all products that carried the government-endorsed traffic light system in the first two years of implementation were SOBF from three supermarket chains [49]. Supermarkets in other countries have implemented nutrient profiling schemes to guide healthy food choice on shelf-edge labels [50], and set targets for the amount of healthy foods sold [51].

Supermarkets have a powerful position in the Australian food system [52] and their decision to support the HSR is significant. Specific examination of uptake of FOPNL on SOBF, and alignment with patterns of nutritional quality are therefore warranted. Assessing FOPNL present on SOBF is important to monitor ongoing implementation of HSR. Alignment of HSR scores on SOBF with the national food selection guide (Australian Guide to Healthy Eating (AGTHE) [12]) can inform the likely impact of the labelling system on public health, given their leadership in HSR implementation and market share. Application of the NOVA classification of level of food processing [53] adds to the analysis of alignment between systems used to measure nutritional quality of foods and HSR. 

This study aimed to address three research questions: (1) What is the prevalence of nutrition labels on the front-of-pack of Australian SOBF? (2) How do Australian SOBF rate for nutritional quality using three different measures: the AGTHE (food group-based), NOVA (food processing-based), and HSR (nutrient-based)? (3) Are Australian supermarkets using the HSR to promote nutritious or nutrient-poor own brand foods?

## 2. Materials and Methods

This study provides a ‘moment-in-time’ examination of SOBF in Perth, Western Australia, including prevalence of the DIG and HSR on SOBF, the HSR scores present, and the nutritional quality of SOBF displaying and not displaying the HSR. Alignment of the HSR with other measures of nutritional quality is also analysed (Supplementary Figure S2).

### 2.1. Selection of Supermarkets

Supermarket audits were conducted in one of each major supermarket chain present in Western Australia, i.e., Coles Supermarkets Australia Pty Ltd. (Coles, Melbourne, Australia), Woolworths Supermarkets (Woolworths, Sydney, Australia), and Independent Grocery Association Supermarkets (IGA, Perth, Australia). Aldi was excluded from this audit due to the different nature of the retail outlets, whereby a limited range of mainly SOBF are sold at discounted prices [54]. The selected supermarkets were conveniently located in Perth in Western Australia, and were large stores with an increased likelihood of displaying most of the SOBF available. The selected Woolworths ‘next generation’ store had been recently extensively refurbished [55]. The selected IGA was an ‘IGA store of the year’ for Western Australia. The selected Coles was the nearest large store to the parent company Wesfarmers’ offices.

### 2.2. Identification of Supermarket Own Brand Foods

Supermarket own brands were identified by use of the supermarket’s branding on the front-of-pack, and by referring to the supermarkets’ websites [56,57]. All packaged foods and non-alcoholic beverages (referred to simply as ‘food’ hereon in) carrying a supermarket own brand were included in the supermarket audits, including packaged unprocessed fresh food such as fruit, vegetables, and meat. The following foods were collected during the supermarket audits, but excluded from this study as the HSR is not an appropriate guide to selection: infant formula, infant food, baking ingredients (e.g., baking powder), culinary condiments (e.g., dried herbs and spices, salt, vinegar) plain coffee, and tea.

### 2.3. Data Collection

Two researchers visited each of the three stores together during a three-week period commencing in February 2017, to conduct audits of SOBF. The main purpose of the audits was to assess the nature and extent of SOBF in Australia, including products available, price, placement, promotion, and nutritional quality. Therefore, data collection involved taking photographic images of the front-of-pack, the shelf-edge label that displayed the price, the location of the product within the store and on the shelf, and any promotional material present. Quality control procedures were implemented to ensure the photographic images captured all the required information for all SOBF present. Photographs were uploaded regularly to a laptop computer and checked for legibility at the end of each day. Any illegible photographs that could not be used were listed and retaken during subsequent visits.

### 2.4. Front-of-Pack Data Extraction 

Photographic images were filed electronically. Relevant details were extracted from the images into Excel databases created for each of the supermarkets. Within each supermarket’s spreadsheet, 18 worksheets were created to capture the information for each product group. Product groups were designated based on the layout of the stores audited, where similar foods were co-located. Within each product group (e.g., bakery and desserts), food groups were created (e.g., biscuits, cakes). Pre-coded responses were established for each column for consistency of data entry. Free text was only permitted for information such as the product name and description. Data entry for the first food group was piloted to ensure all necessary information was entered and to establish any final pre-coding changes needed. Two researchers conducted data extraction from the photographic images. Both researchers reviewed the data for accuracy and changes were implemented by the first author as required to ensure consistency of approach.

The data extracted from the photographic images included information displayed on the front-of-pack, such as supermarket own brand, product name and description, pack weight or volume, and voluntary nutrition labels. The FOPNL identified in this audit included: (a) the HSR only; (b) the HSR plus kilojoules per 100 g; (c) the HSR plus kilojoules, saturated fat, sugars, sodium per 100 g and an optional nutrient; (d) the HSR energy only icon [58]; (e) the DIG thumbnail icon which displays kilojoules per serve; and (f) the DIG preferred format of kilojoules, fat, saturated fat, sugars and sodium per serve [15].

### 2.5. Assessment of Nutritional Quality 

The nutritional quality of all SOBF present was assessed using front-of-pack information only. Nutritional quality was assessed using the recommendations of the AGTHE, which identifies nutritious foods which are part of the recommended five food groups, and energy-dense-nutrient-poor or ‘discretionary’ foods which should be limited [12]. The NOVA classification of level of food processing, which aims to address the impact of industrial food processing on health, was also applied [53]. 

The HSR provided on the front-of-pack was recorded as displayed, and was not calculated for products where it was not present. Although the HSR is not intended to be used on fruit, vegetables, meat, poultry and fish, they are not excluded [15], therefore these products were not excluded from the analysis. A HSR of 2.0 or less was taken to be an appropriate cut-off for nutrient-poor foods, and a HSR of 2.5 or more appropriate for nutritious foods [40]. The use of 2.5 stars as a ‘pass’ rating is logical and has more credibility as a potential consumer education message than use of 3.5 stars [40]. For example, consumers could be advised that foods with HSR of at least 2.5 stars are more likely to be a nutritious choice. It is not logical to have a system which attributes 3 stars out of a possible 5 stars to a nutrient-poor food that is not consistent with the AGTHE and expect consumers to deduce it would be a poor food choice. It is also not consistent with dietary guideline recommendations to discourage consumption of nutrient-poor discretionary foods. Qualitative research has confirmed that consumers tended to use the HSR in a binary way, categorising foods with HSR of 2 stars or less as unhealthy, and foods with HSR of 3 stars or more as healthier [35].

The AGTHE nutritious five food groups included: vegetables, legumes and beans; fruit; grain or cereal foods; lean meat, poultry, fish, eggs, tofu, nuts, and seeds; and milk, yoghurt, cheese, and their alternatives. Nutrient-poor discretionary foods include items that are high in saturated fat, sugars, salt, or alcohol. Examples are provided in the Educator’s Guide [42]; however, they are limited to whole foods, not meals or mixed foods, and provide overarching principles that can be applied to dietary analysis more easily than packaged food classification. The Australian Bureau of Statistics (ABS) has established principles for identifying discretionary foods [41]. The ABS principles were adapted for this study, as there were many ready-to-eat products present in the audit which were not addressed by the ABS criteria, and product nutrition information was not available to inform classification. A decision tree was constructed to enable classification of products in accordance with the recommendations of the AGTHE, with the addition of two new food groups: ‘Mixed products using mainly five food group foods’, and ‘Mixed products high in fat, salt or sugar’ (Supplementary Table S1).

The NOVA classifications included: unprocessed or minimally processed foods (e.g., fruit, vegetables, meat, grains, nuts); processed culinary ingredients (e.g., salt, sugar, vegetable oils, butter); processed foods which are simple foods made with few ingredients (e.g., canned vegetables, canned fish, cheese, cured or smoked meat); and ultra-processed foods (UPF), which are nutrient-poor, industrial formulations that include ingredients or processes not found in the home (e.g., savoury snacks, cereal bars, biscuits, instant sauces, pre-prepared dishes such as pies and pizzas) [53]. Studies have shown UPF have higher saturated fat, sugar and sodium content compared to less processed foods [53,59]. High levels of UPF consumption have been associated with excess weight [60], increased risk of cancer [61], and reduced diet quality [62]. The recommendations of the AGTHE and the HSR algorithm do not currently consider the level of food processing; however, researchers have identified the benefits of incorporating consideration of the level of food processing into national policies to improve dietary health [63].

### 2.6. Statistical Analysis

Data were analysed using the SPSS for Windows statistical software package version 24 (IBM Corp. Released 2016. Armonk, NY, USA: IBM Corp USA). The frequency of use of six different formats of FOPNL was compared between the three supermarkets. A comparison of the frequency of HSR labels on foods classified using the AGTHE food groups, and the NOVA levels of food processing was produced. For SOBF displaying the HSR, mean HSR, standard deviation, minimum, and maximum were derived for all AGTHE food groups and foods classified using the NOVA levels of food processing. Charts that displayed the frequency of HSR scores by supermarket chain, AGTHE food group, and NOVA level of food processing were prepared. Chi-square tests of independence were performed to examine the relationship between presence of HSR on the front-of-pack of SOBF and their nutritional quality as assessed using the AGTHE and NOVA, and to examine the relationship between foods that achieved a HSR of 2.5 or above and HSR of 2.0 and below and their nutritional quality as assessed using the AGTHE and NOVA. 

## 3. Results

Approximately 20,000 photographic images were collected for 3940 SOBF in this audit. There were 1812 SOBF present in the Woolworths store, 1731 SOBF in the Coles store, and 397 SOBF in the IGA store. After excluding infant formula, infant food, baking ingredients, culinary condiments, plain coffee, and tea, there were 3737 SOBF included in this study: 1707 from Woolworths, 1645 from Coles, and 385 from IGA (Table 2).

### 3.1. Prevalence of Front-of-Pack Nutrition Labels on Supermarket Own Brand Foods

Most SOBF (81.5%) featured either the HSR or DIG on the front-of-pack (Table 2), no products included both. Over half of all SOBF (55.1%) featured the HSR. Coles had the largest proportion of foods featuring the HSR (69.2%), followed by Woolworths (54.0%), and the HSR was not present on any SOBF in IGA. The full HSR logo that includes kilojoules and nutrient information was the most commonly used version across Coles and Woolworths, present on 33.0% of all audited products. A quarter of SOBF (26.4%) featured the DIG. The DIG was present on most IGA SOBF (81.0%), 26.9% of Woolworths, and 13.0% of Coles SOBF.

Analysis of presence of FOPNL on SOBF included packaged unprocessed fresh food such as fruit, vegetables, and meat (*n* = 438), even though the HSR was not intended to be used on such foods. HSR was present on 4.1% of all packaged fresh foods, including: fish, beef, pork, lamb, vegetables. DIG was present on 2.7% of all packaged fresh foods, including: beef, pork, lamb, herbs, chicken and vegetables.

### 3.2. Nutritional Quality of Supermarket Own Brand Foods Using the Australian Guide to Healthy Eating

Using the principles of the AGTHE, half (51.3%) of the SOBF present in this study were classified as nutritious foods, 46.6% were nutrient-poor, and 2.1% were culinary ingredients (e.g., mustard, liquid stock) (Table 3). The nutritious food group with the most SOBF present was the meat and meat substitute group which included lean meat, fish, eggs, tofu, nuts and seeds (14.0%), followed by grain or cereal foods (13.0%). However, the proportion of nutrient-poor discretionary SOBF present was far greater at 45.2%.

### 3.3. Nutritional Quality of Supermarket Own Brand Foods Using NOVA 

Over half (56.9%) of all SOBF were classified as UPF (Table 3). A quarter (24.8%) of SOBF were unprocessed or minimally processed, 15.1% were processed foods, and 3.2% were processed culinary ingredients. 

### 3.4. Nutritional Quality of Supermarket Own Brand Foods Using HSR Scores 

**Figure 1:**
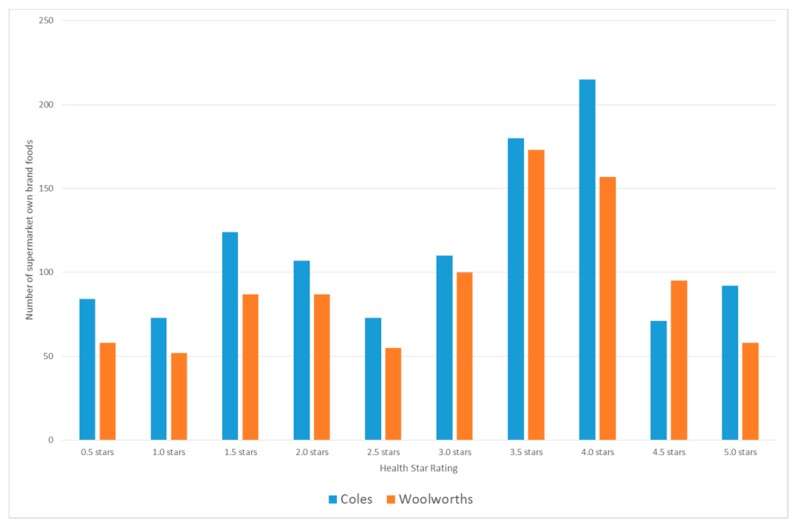
Frequency of Health Star Rating scores for supermarket own brand foods.

The HSR was not calculated for foods that did not display the device (*n* = 1688), so the dataset used for analysis includes 2049 SOBF. The mean HSR of all SOBF was 2.96 (range 0.5–5.0, *n* = 2049). The mean HSR for Coles SOBF was 2.92 (range 0.5–5.0, *n* = 1129), and the mean HSR for Woolworths SOBF was 3.01 (range 0.5–5.0, *n* = 921). The most frequently occurring HSR scores were 3.5 stars (Coles *n* = 180, Woolworths *n* = 173) and 4.0 stars (Coles *n* = 215, Woolworths *n* = 157) (Figure 1). More of the Woolworths SOBF scored HSR of ≥2.5 compared to Coles SOBF (69.1% for Woolworths, 65.7% for Coles).

### 3.5. Alignment between HSR and Other Measures of Nutritional Quality 

**Figure 2:**
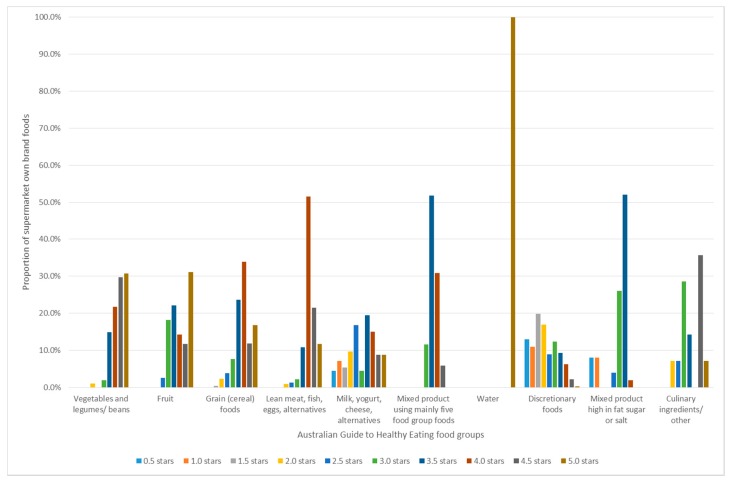
Frequency of Health Star Rating scores for supermarket own brand foods classified using the principles of the Australian Guide to Healthy Eating.

Supermarket own brand food groups classified as nutritious using the AGTHE achieved a range of mean HSR scores (Table 3). Mean HSR scores for nutritious food groups were all above the designated cut-off of 2.5 stars. Vegetables, legumes and beans had a mean HSR of 4.3; fruit had a mean HSR of 4.0; grain or cereal foods had a mean HSR of 3.9; lean meat, fish, eggs tofu, nuts, and seeds had a mean HSR of 4.1; milk, yogurt, cheese, and alternatives had a mean HSR of 3.0; and mixed foods using mainly five food group foods had a mean HSR of 3.7. However, Figure 2 shows the frequency of HSR scores for each food group; 26.5% of the milk, yogurt, cheese, and alternatives food group scored below 2.5 stars. Nutrient-poor SOBF failed to achieve mean HSR scores of the designated cut-off of 2.0 or below. Discretionary foods had a mean HSR of 2.1; and mixed foods high in fat sugar or salt had a mean HSR of 2.9. Figure 2 shows the frequency of HSR scores for these nutrient-poor foods; 39% of discretionary foods and 84% of mixed products high in fat sugar or salt scored HSR of 2.5 or over.

**Figure 3:**
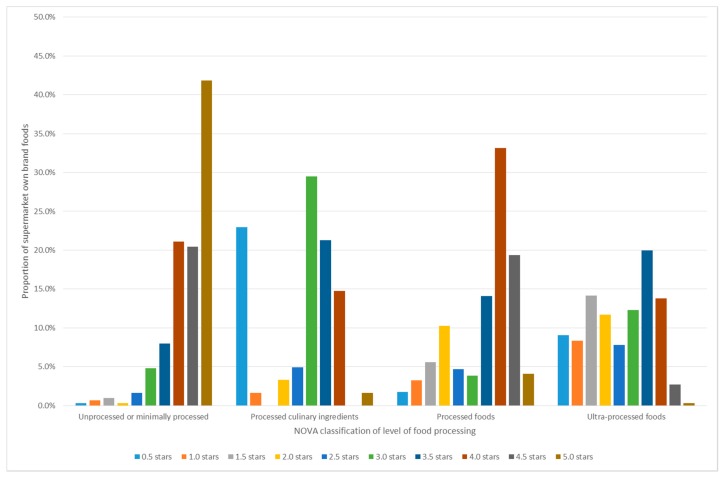
Frequency of Health Star Rating scores for supermarket own brand foods classified using the NOVA level of food processing.

The food groups recommended in the NOVA classification system as the foundation of healthy dietary patterns, unprocessed and minimally processed foods, achieved a mean HSR of 4.4 (Table 3). Processed foods achieved a mean HSR of 3.5, and processed culinary ingredients achieved a mean HSR of 2.6. The food group recommended to be avoided in the NOVA classification system, nutrient-poor UPF, achieved a mean HSR of 2.5. Therefore, nutrient-poor ultra-processed SOBF failed to meet the designated HSR cut-off of 2.0 or below. Figure 3 shows the frequency of HSR scores for the NOVA food groups: 98% of nutritious unprocessed or minimally processed foods scored HSR of 2.5 or over; however, 55% of nutrient-poor UPF also scored HSR of 2.5 or over.

### 3.6. Presence of HSR on Nutritious and Nutrient-Poor Supermarket Own Brand Foods

A chi-square test of independence was performed to examine the relationship between presence of HSR on the front-of-pack of SOBF and their nutritional quality (Table 4). Using the AGTHE food group classifications, nutrient-poor SOBF were more likely to display HSR than nutritious foods. Using the NOVA classification of level of food processing, nutrient-poor UPF were more likely to display HSR than other foods.

A chi-square test of independence examined the relationship between foods that achieved a HSR of 2.5 or above (an appropriate score for nutritious foods) and HSR of 2.0 and below (an appropriate score for nutrient-poor foods) and other measures of nutritional quality (Table 5). Foods classified as nutritious using the principles of the AGTHE were more likely to display HSR ≥ 2.5 than nutrient-poor foods. However, foods classified as nutrient-poor UPF using NOVA were more likely to achieve a HSR ≥ 2.5 than all other foods. In addition, the results indicate that of the SOBF carrying a HSR label, 41.3% of AGTHE nutrient-poor foods and 4.2% of AGTHE nutritious foods were given inappropriate HSR scores.

## 4. Discussion

Supermarkets play a powerful gatekeeper role in the Australian food system [52] and are key supporters of the government-led front-of-pack HSR system [45]. This study aimed to examine prevalence of the HSR and food industry-led DIG on SOBF in Australia. It also aimed to assess the nutritional quality of SOBF using the AGTHE [12] and NOVA levels of food processing [53], and identify whether uptake of the HSR was aligned with these measures.

This study found that most SOBF in Australia included a FOPNL of some kind. HSR was widespread on Coles SOBF, present on over half of Woolworths SOBF, but not present on any IGA SOBF, which used the DIG instead. IGA supermarkets are independently operated and predominantly supplied with products by Australia’s largest wholesaler Metcash (Sydney, Australia), who is also responsible for the marketing of IGA [64]. The decision not to add HSR to SOBF was therefore made by Metcash. In February 2018, Metcash stated they used the DIG instead of HSR as they believed it was “*more beneficial for shoppers… due to the increased nutritional information it provides*” [65]. It is interesting to note that Metcash is a member of the government-led Healthy Food Partnership (HFP) [66], which advocates use of the HSR as a key support to their work on product reformulation [67]. As HSR and the HFP are the only two Australian government initiatives which aim to address population dietary health, lack of HSR uptake by a HFP member deserves further examination.

Findings indicate that while good progress had been made in applying the HSR to SOBF, neither Coles nor Woolworths had fulfilled their commitments to label all SOBF with the HSR by 2016 [45]. This is important, because the two supermarkets privately govern the Australian food system [52], and failure to fulfil commitments may erode trust in their ability. Only a third of SOBF from the bread and alternatives product group displayed either the HSR or DIG. However, many of these products were from the in-store bakery, which carried labels with branding, ingredients and allergen information only. All foods carrying a supermarket own brand on the label were included in this study, including packaged fresh whole foods such as fruit, vegetables, fish and meat, which the HSR style guide states the system was not intended for [58]. However, few of the prepacked fresh foods displayed either the HSR or DIG. The only foods excluded from analysis were infant formula, infant food, baking ingredients, culinary condiments, plain coffee, and tea, as the HSR cannot usefully guide selection of these items. The HSR system is currently undergoing a five-year review, and one of the areas of consideration is the foods it should appear on [68]. Given an automatic five-star rating currently applies to packaged water [58], which is a product not required to carry nutrition information [69], and the fact that supermarkets are currently displaying the HSR on selected fresh whole foods such as meat, it is logical to encourage its use across packaged whole foods, especially the nutritious foods recommended in the AGTHE [12]. Scoring of these recommended nutritious whole foods needs to be considered, one option being allocation of the same automatic five-star rating as water. In addition, consideration of applying the HSR to shelf-edge tags for unpackaged or fresh whole foods, similar to the American Guiding Stars system [70], should be considered.

An assessment of the proportion of packaged products displaying the HSR in Sydney supermarkets in 2017 [31] had some differences in findings to this assessment of SOBF in Perth supermarkets. There were more SOBF identified in the Perth stores, which is only partly explained by the inclusion of packaged whole fresh foods that carried an own brand on the label. Some of these foods, including packaged fresh meat, displayed the HSR which was the reason for their inclusion. There were more Coles SOBF foods which carried the HSR on labels identified in the Sydney audit (1246 versus 1128), which could be a result of differences in the products sold between locations, or the timing of the audits. The differences were more pronounced when comparing findings for Woolworths. The Perth audit identified considerably more Woolworths SOBF foods which carried the HSR (921 versus 713); however, the greater number of SOBF overall meant that the proportion reported in this study was significantly lower compared to the Sydney-based study [31]. Similarly, there were considerable differences in the IGA findings. More than double the number of SOBF were identified in the Perth audit compared to the Sydney audit. IGA supermarkets are not centrally managed, and available products are selected by the owner or manager. Therefore, the difference in the number of SOBF is not surprising. Studies found no (or one) IGA SOBF carried the HSR. These differences in findings for the number of SOBF present, and the proportion displaying the HSR, indicate that examination of differences in product availability between the Australian States and Territories is needed. This is important because most Australian studies assessing attributes of supermarket environments to date have been conducted in Sydney and Melbourne [71], and findings may not translate to other metropolitan areas within Australia. 

The nutritional quality of SOBF identified in this study was assessed using three different measures: the AGTHE [12], NOVA [53], and HSR [72]. Most Australians do not eat the recommended amount of nutritious foods needed for good health, and a third of population energy intake was from nutrient-poor discretionary foods in 2011–12 [73]. Only half of the SOBF present were classified as nutritious foods recommended by the AGTHE. This proportion is higher than that of a recent evaluation of the nutritional quality of the Australian food supply [74], only partly explained by the inclusion of packaged fresh whole foods in this study. Over half of the SOBF present were classified as nutrient-poor UPF. This is consistent with other studies that have found a large proportion of such highly processed foods present in the Australian food supply [74,75]. In fact, most new products launched in Australia in 2015 were UPF [76]. Supermarkets control development of SOBF by suppliers and can determine the nutritional quality [77]. They can also use SOBF to influence consumer food choice [78]. This study shows that more effort is needed by Australian supermarkets to ensure their contribution to the food supply supports healthy food selection. 

A range of mean HSR scores were obtained for each of the AGTHE nutritious five food groups in this study. Three of the five food groups achieved a mean HSR above 4 stars, and few of these foods scored less than 2.5 stars. However, of the recommended dairy foods with HSR present, a quarter failed to be scored as nutritious. Previous investigation of the ability of HSR to identify nutritious dairy foods also found a large proportion scored less than 2.5 stars, particularly hard cheeses [79]. The authors attributed this finding to the contribution of saturated fat content to the HSR algorithm [79]. Technical flaws in the ability of the HSR algorithm to identify recommended dairy foods have also been identified by Lawrence et al. [40]. The mean HSR scores for nutrient-poor discretionary foods, mixed products high in fat sugar or salt, and UPF, and the predominance of UPF with HSR scores indicating they were nutritious choices, further illuminate the inconsistencies between HSR and other measures of nutritional quality. This study’s findings indicate the HSR algorithm currently fails to score recommended dairy foods, discretionary foods, mixed products high in fat sugar or salt, or UPF appropriately. 

The French FOPNL was also based on the UK nutrient profiling model used by Ofcom to regulate food advertising to children [26]. Unlike the HSR, the French Nutri-Score label underwent analysis for consistency with the French nutritional recommendations, whereby adaptations were made to the algorithm to improve the ability of the system to discriminate between recommended and nutrient-poor foods [26]. In Australia, a wide variety of nutritious five food group foods are recommended [42]. Advice is also given on the amount to eat and best choices to make, for example: choose canned legumes and vegetables without added salt, whole fruit is preferable to fruit juice, wholegrains are preferable to refined grains, no more than 455g of cooked lean red meat is recommended each week, low and reduced-fat dairy foods are better choices for most people compared to full-fat dairy foods [42]. Adapting the algorithm to ensure it promotes the recommendations of the Australian Dietary Guidelines using the same three levels of detail examined in the French study [26] is recommended: across food groups (i.e., nutritious and nutrient-poor food groups obtain scores that are demarcated), within food groups (i.e., best choices and all other choices obtain scores that are demarcated), and similar products from different brands obtain scores that allow for meaningful comparison.

Several studies have assessed the alignment of FOPNL with dietary guidelines, and there is a current lack of consistency in the cut-off scores assigned to identify nutritious and nutrient-poor foods. For example, the French study that assessed the ability of the Nutri-Score label to discriminate nutritional quality stated that foods to be encouraged (i.e., nutritious foods) should have a green or yellow rating, and foods to be limited (i.e., nutrient-poor or energy-dense foods) should have a pink or red rating; the midway rating of orange was not applied to either group [26]. Applying the same cut-off principles to the HSR would translate to nutritious foods attaining ≥3.5 stars, and nutrient-poor foods attaining ≤2.0 stars. One Australian study assigned cut-off scores to identify HSR that were ‘*apparent outliers’*, stating nutrient-poor foods should not score ≥3.5 stars, and nutritious foods should not score ≤2.0 stars [39]. Other Australian studies have used HSR of 3.5 as a cut-off to distinguish between foods that are recommended in the dietary guidelines, and nutrient-poor discretionary foods [29,31,74,80]. They refer to work which analysed the alignment of the HSR with the colour-coded Traffic Light system used by the New South Wales Government to identify nutritious foods in settings such as schools, hospitals, and workplaces [43]. Another Australian study used HSR of ≥2.5 to indicate scores that are appropriate for nutritious foods [40]. Given FOPNL is a highly contested area of food labelling [2], and selection of HSR scores considered appropriate for nutritious and nutrient-poor foods can impact findings, robust and transparent analysis of the implications of HSR cut-off scores is recommended.

The current study’s findings indicate that application of HSR by two supermarkets on SOBF has served to promote nutrient-poor food choices. Nutrient-poor and ultra-processed SOBF were more likely to include the HSR on the front-of-pack than nutritious foods, and many achieved HSR scores of 2.5 stars and over, inaccurately indicating they were a healthy choice. These findings are likely to reflect flaws inherent in the system which are currently being considered in a five-year review [68], rather than supermarket decision-making *per se*. Decisions made by the HSR advisory committee to exclude foods such as packaged fruit, vegetables, meat, and fish from the expectation of HSR labelling, but assign an automatic five stars to packaged water [58], means the recommendations of the Australian Dietary Guidelines [12] are not promoted consistently. Specific concerns about the ability of the HSR algorithm to promote recommended nutritious foods have also been raised [40,81,82,83,84]. Regardless, Australia’s two largest supermarkets [44], which wield enormous power over the food system [52], have been key supporters of the HSR, leading uptake on packaged food [31]. Coles and Woolworths have participated in Australian government-led population nutrition initiatives such as HSR and the HFP since 2009, but their influence is unknown [52]. In particular, lack of transparency over development, validation, and implementation of HSR in Australia means the drivers of decision-making remain hidden. Research to determine the nature and extent of influence by supermarkets, and others with vested interests, over decisions that affect national nutrition policy (i.e., HSR and HFP) is needed.

Strengths of this study include the large sample size, and the specific focus on examining use of FOPNL on SOBF which was driven by the commitment of two supermarkets to adopt the HSR across all SOBF. In addition, the supermarket audit methodology collected data over three weeks from three large stores, which were purposively selected to give an increased likelihood of most of the SOBF being available. The extensive data collection conducted over a short time period meant that only front-of-pack photographs were taken. Therefore, the ABS discretionary food list, which uses nutrient cut-offs in some of its definitions, was not suitable for classification of foods consistent with the recommendations of the AGTHE in this study. However, a rigorous process was developed to ensure consistency in using front-of-pack information for classification, and some products displayed the full HSR label, which includes nutrient information. Data were collected in Perth in Western Australia, and findings may not translate to other Australian metropolitan areas, as consistency in SOBF availability is currently not known. Seasonality may also affect findings, and as this study was conducted between the end of the Australian school summer holidays and Easter, supermarket audits conducted at other times of the year may find different SOBF availability.

## 5. Conclusions

This study found that most SOBF present in three Perth supermarkets included a FOPNL. HSR application was widespread on the Coles SOBF, present on over half of the Woolworths SOBF, but not present on any of the IGA SOBF, which used the DIG instead. Nutrient-poor and ultra-processed SOBF were more likely to include the HSR on the front-of-pack than nutritious foods, and many of these foods achieved HSR scores indicating they were a healthy choice. Supermarkets have a powerful position in the Australian food system, and they could do more to support healthy food selection. Recommendations for supermarkets include:

(i) Use their influence and power by advocating to government for changes to the HSR algorithm, to ensure it achieves the original policy aim of identifying healthier foods consistent with the Australian Dietary Guidelines;
(ii) Apply the HSR to all foods including packaged unprocessed fresh foods such as fruit, vegetables, fish and meat;
(iii) After the algorithm has been modified to ensure it achieves the original policy aim, Coles and Woolworths should fulfil their commitments to label all SOBF with the HSR and remove the DIG from packaging. Metcash should support application of HSR to all SOBF and remove the DIG from packaging;
(iv) Consider setting targets to improve the proportion of SOBF that are classified as nutritious using the AGTHE, NOVA, or HSR score; and
(v) Increase transparency of contributions to key government-led initiatives that aim to improve the dietary health of all Australians (i.e., HSR and HFP).

In addition, future research recommendations include:

(i) Compare differences in SOBF availability between the Australian States and Territories, to determine whether supermarket audit findings can be translated between metropolitan regions.
(ii) Adapt the HSR algorithm to ensure it promotes the recommendations of the Australian Dietary Guidelines using three levels of detail: across food groups (i.e., nutritious and nutrient-poor food groups obtain scores that are demarcated), within food groups (i.e., best choices and all other choices obtain scores that are demarcated), and similar products from different brands obtain scores that allow for meaningful comparison. In particular, this study’s findings indicate the HSR algorithm currently fails to score nutritious dairy foods, nutrient-poor discretionary foods, mixed products high in fat sugar or salt, or UPF appropriately.
(iii) Assess and report on the nature and extent of supermarket (i.e., Coles and Woolworths), and wholesaler (i.e., Metcash) influence over decisions that affect Australian food and nutrition policy, by analysing their contribution to HSR and the HFP.

## Figures and Tables

**Table 1:** Methodological decisions that can influence study findings on whether HSR product scores are consistent with recommendations of the Australian Dietary Guidelines.

Methodological Decision	Study 1 [39]	Study 2 [40]
Source of data	The George Institute for Global Health’s Australian FoodSwitchDatabase, which conducts annual surveys and receives data from manufacturers and consumers	The Mintel Global New Products Database which collects packaging data and images of all new packaged foods launched in Australia and New Zealand
Date	1 January 2013–30 June 2017	27 June 2014–30 June 2017
Number of products included in analysis	*n* = 65,660	*n* = 1269
HSR product score	Calculated from nutrition information present on pack, and proxy values were estimated for missing values (e.g., fruit, vegetable, nut, legume, or fibre content which are not required on labels)	Extracted from packaging photographic images, not calculated for products with no HSR displayed
Classification of products consistent with the recommendations of the Australian Dietary Guidelines	Classification of recommended nutritious foods was informed by the Australian Dietary Guidelines [12]; classification of nutrient-poor foods utilised the Australian Bureau of Statistic’s Discretionary Food List [41].	Classification of recommended nutritious foods was informed by the Australian Dietary Guidelines Educator’s Guide [42]; classification of nutrient-poor foods utilised the Australian Bureau of Statistic’s Principles for Identifying Discretionary Foods, and the Discretionary Food List [41].Products difficult to classify were coded by each author individually and then a consensus decision made.
Determination of HSR scores consistent with the Australian Dietary Guidelines	No justification provided. However, a study which analysed alignment of the HSR with the Traffic Light system used by the New South Wales Government to identify nutritious foods in settings such as schools, hospitals, and workplaces recommended that foods with HSR ≥ 3.5 were more likely to be ‘green’ or nutritious [43].	A HSR of 2.5 was deemed to be a ‘pass’ rating appropriate for nutritious foods; a HSR of 2.0 or lower was deemed to be a ‘fail’ rating appropriate for nutrient-poor foods.
HSR demarcation of recommended nutritious foods and nutrient-poor foods	Nutritious foods should not have a HSR ≤ 2.0Nutrient-poor foods should not have a HSR ≥ 3.5	Nutritious foods should not have a HSR ≤ 2.0Nutrient-poor foods should not have a HSR ≥ 2.5

HSR is Health Star Rating.

**Table 2:** Front-of-pack nutrition labels present on supermarket own brand foods in Australia.

	Coles		Woolworths		IGA		All Supermarkets	
	Frequency	Percent	Frequency	Percent	Frequency	Percent	Frequency	Percent
Health Star Rating with kJ and nutrients	662	40.2%	570	33.4%	0	0.0%	1232	33.0%
Health Star Rating with kJ	149	9.1%	118	6.9%	0	0.0%	267	7.1%
Health Star Rating only	318	19.3%	233	13.6%	0	0.0%	551	14.7%
Health Star Rating energy only icon	9	0.5%	0	0.0%	0	0.0%	9	0.2%
*Sub-total: Health Star Rating present*	*1138*	*69.2%*	*921*	*54.0%*	*0*	*0.0%*	*2059*	*55.1%*
Daily Intake Guide with kJ and nutrients	185	11.2%	408	23.9%	159	41.3%	752	20.1%
Daily Intake Guide kJ only	29	1.8%	52	3.0%	153	39.7%	234	6.3%
*Sub-total: Daily Intake Guide present*	*214*	*13.0%*	*460*	*26.9%*	*312*	*81.0%*	*986*	*26.4%*
*Total: Front-of-pack nutrition labels present*	*1352*	*82.2%*	*1381*	*80.9%*	*312*	*81.0%*	*3045*	*81.5%*
**Total**	**1645**		**1707**		**385**		**3737**	

**Table 3:** Health Star Rating scores for supermarket own brand foods in Australia, classified by nutritional quality.

	Supermarket Own Brand Foods Present		Supermarket Own Brand Foods Displaying HSR		Health Star Rating			
	N	%	N	%	Mean	SD	Min.	Max.
**Australian Guide to Healthy Eating food groups**								
Nutritious foods								
Vegetables, legumes and beans	351	9.4	101	4.9	4.34	0.604	2.0	5.0
Fruit	166	4.4	77	3.8	4.04	0.802	2.5	5.0
Grain or cereal foods	484	13.0	263	12.8	3.92	0.727	1.5	5.0
Lean meat, fish, eggs, tofu, nuts and seeds	523	14.0	223	10.9	4.11	0.524	2.0	5.0
Milk, yogurt, cheese, alternatives	185	5.0	113	5.5	3.04	1.258	0.5	5.0
Mixed product using mainly five food group foods	184	4.9	172	8.4	3.65	0.376	3.0	4.5
Water	25	0.7	11	0.5	5.00	0.000	5.0	5.0
*Sub-total: nutritious foods*	*1918*	*51.3*	*960*	*46.9*	*3.88*	*0.806*	*0.5*	*5.0*
Nutrient-poor foods								
Discretionary foods	1689	45.2	1025	50.1	2.09	1.102	0.5	5.0
Mixed product high in fat sugar or salt	52	1.4	50	2.4	2.90	0.995	0.5	4.0
*Sub-total: nutrient-poor foods*	*1741*	*46.6*	*1075*	*52.5*	*2.13*	*1.110*	*0.5*	*5.0*
Other foods								
Culinary ingredients/other	78	2.1	14	0.7	3.64	0.929	2.0	5.0
**NOVA food processing classification**								
Unprocessed or minimally processed	928	24.8	313	15.3	4.35	0.790	0.5	5.0
Processed culinary ingredients	119	3.2	59	2.9	2.62	1.303	0.5	5.0
Processed foods	564	15.1	341	16.6	3.46	1.114	0.5	5.0
Ultra-processed foods	2126	56.9	1336	65.2	2.52	1.178	0.5	5.0
**Total**	**3737**		**2049**	**54.6**	**2.96**	**1.310**	**0.5**	**5.0**

N is number, SD is standard deviation, Min. is minimum, Max. is maximum.

**Table 4:** Chi-square test of independence between presence of HSR on the front-of-pack of supermarket own brand foods and their nutritional quality.

Nutritional Quality	Health Star Rating Present	No Health Star Rating Present	Chi Square Tests of Independence	
	N (Percent)	N (Percent)	χ^2^	*p* Value
**Australian Guide to Healthy Eating classification**				
Nutrient-poor foods	1075 (52.8%)	666 (41.0%)	51.509	<0.001
Nutritious foods	960 (47.2%)	958 (59.0%)		
**NOVA food processing classification**				
Ultra-processed foods	1336 (65.2%)	790 (46.8%)	128.121	<0.001
All other foods	713 (34.8%)	898 (53.2%)		

**Table 5:** Chi-square test of independence between HSR scores and measures of nutritional quality.

Nutritional Quality	Health Star Rating ≤ 2.0	Health Star Rating ≥ 2.5	No Health Star Rating Present	Chi Square Tests of Independence	
	N (Percent)	N (Percent)	N (Percent)	χ^2^	*p* Value
**Australian Guide to Healthy Eating classification**					
Nutrient-poor foods	631 (94.0%)	444 (32.6%)	666 (41.0%)	732.303	<0.001
Nutritious foods	40 (6.0%)	920 (67.4%)	958 (59.0%)		
**NOVA food processing classification**					
Ultra-processed foods	577 (85.9%)	759 (55.1%)	790 (46.8%)	310.828	<0.001
All other foods	95 (14.1%)	618 (44.9%)	898 (53.2%)		

## Supplementary Materials

The following are available online at http://www.mdpi.com/2072-6643/10/10/1465/s1, Table S1: Procedure to classify foods consistent with the Australian Guide to Healthy Eating; Figure S1: Front of pack nutrition labels present in Australia; Figure S2: The research process.
