# Supplementary material for: Alignment of Supermarket Own Brand Foods’ Front-of-Pack Nutrition Labelling with Measures of Nutritional Quality: An Australian Perspective
Source: Nutrients. 2018 Oct 9;10(10):1465. doi: 10.3390/nu10101465 (PMC6213021; doi:10.3390/nu10101465)
Supplement: Supplementary file 1 [file nutrients-10-01465-s001.zip › suppl/Supplementary figure 1.pdf]

Supplementary Figure 1: Front of pack nutrition labels present in Australia

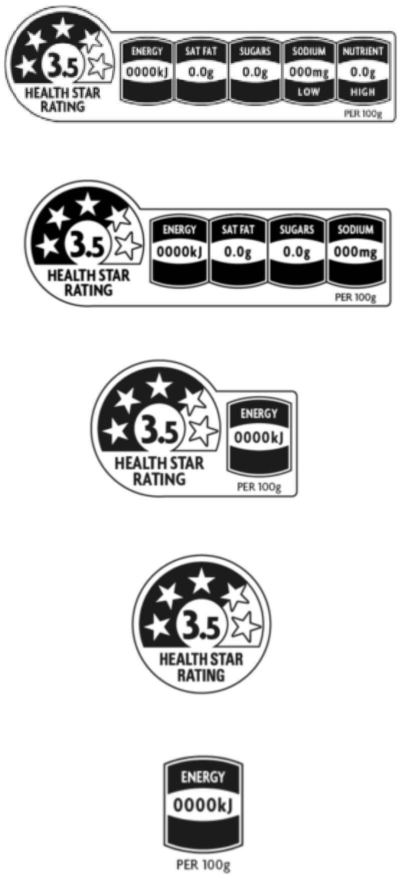<br>**The different options for displaying the Health Star Rating system graphic**	**Positive attributes:**<br>Protein<br>Dietary fibre<br>Fruit, vegetable, nut and legume content<br>Calcium – for dairy beverages and dairy foods<br>**Negative attributes:**<br>Energy<br>Saturated fat<br>Total sugar<br>Sodium<br>**Nutrients and ingredients that are included in the Health Star Rating algorithm**
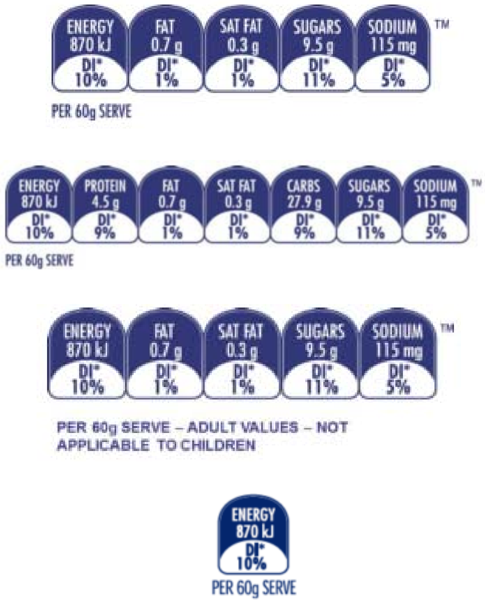<br>**The options for displaying the Daily Intake Guide**	**Core nutrients:**<br>Fat<br>Saturated fat<br>Total sugar<br>Sodium<br>**Optional nutrients:**<br>Protein<br>Carbohydrate<br>**Nutrients recommended for display in the Daily Intake Guide**
