# Supplementary material for: Alignment of Supermarket Own Brand Foods’ Front-of-Pack Nutrition Labelling with Measures of Nutritional Quality: An Australian Perspective
Source: Nutrients. 2018 Oct 9;10(10):1465. doi: 10.3390/nu10101465 (PMC6213021; doi:10.3390/nu10101465)
Supplement: Supplementary file 1 [file nutrients-10-01465-s001.zip › suppl/Supplementary Table 1.docx]

Supplementary Table 1: Procedure to classify foods consistent with the Australian Guide to Healthy Eating

| **Question** | **Details** | **If yes…** | **If no or unsure…** |
| --- | --- | --- | --- |
| Q1. Is the product easily identifiable as a five food group food, or water? | Vegetables - All fresh, frozen, canned and dried, but not fried | Classify into the appropriate food group | Go to Q2 |
|  | Fruit - All fresh, frozen, canned, dried, and fruit juice |  |  |
|  | Grains - Whole and rolled grains, flour, bread, pasta, noodles, breakfast cereals, including refined and whole grain varieties |  |  |
|  | Lean meat, fish, and alternatives - All fresh, frozen and canned meat, poultry and fish; salt and fat reduced sausages; eggs, tofu, nuts and nut spreads, legumes, seeds |  |  |
|  | Milk, yoghurt, cheese, and alternatives - Fresh, dried, evaporated or UHT milk, yoghurt, all cheese, and calcium-enriched alternatives |  |  |
|  | Water |  |  |
| Q2. Is the product easily identifiable as a discretionary food, using the examples provided in the Eat for Health Educators Guide? | Foods with higher added sugars - energy drinks, fruit drinks, honey, jams, marmalade, some sauces, sports drinks, sugar, confectionery, soft drinks, cordials, sweetened waters, iced tea, syrups | Classify as discretionary | Go to Q3 |
|  | Foods with higher saturated fat - bacon, ham, butter, cream, ghee, some tacos/nachos/enchiladas, commercially fried foods, commercial burgers, crisps, extruded snacks, dairy blends, frankfurts, chips, meat pie, pasties, pastry, pizza, processed meat, quiche, salami, mettwurst, sausages, some crackers, some sauces, spring roll |  |  |
|  | Foods with higher saturated fat and added sugars - biscuits, cakes, chocolate, chocolate bars, dessert style custards, doughnuts, iced buns, ice cream, muesli bars, puddings, slices, some confectionery, some sauces, muffins, pastries, pies, crumbles |  |  |
|  | Foods with high salt - marinades and sauces e.g. fish sauce, soy sauce; salty snack foods; spreads e.g. Vegemite; savoury biscuits |  |  |
| Q3. Do the ABS principles for identifying discretionary foods identify this food as discretionary? | All milk drinks including flavoured milk | Classify as milk, yoghurt, cheese and alternatives | Go to Q4 |
|  | All soft drinks including those with intense sweeteners | Classify as discretionary |  |
|  | All fruit drinks other than fruit juices |  |  |
|  | Tea or coffee with added sugar |  |  |
|  | Breakfast cereals without added fruit > 30g sugar/100g |  |  |
|  | Breakfast cereals with added fruit > 35g sugar/100g |  |  |
|  | All dry soup mixes |  |  |
|  | Mixed dishes containing grains e.g. sandwiches, burgers, wraps, sushi, pizza >5g saturated fat/100g | Classify as ‘mixed product high in fat salt or sugar’ |  |
| Q4. Does the product contain any of the following: added saturated fat, added salt, or added sugar? | added saturated fat e.g. butter, cream, coconut milk/cream, mayonnaise | Go to Q5 | Classify as 'mixed product using mainly five food group foods' |
|  | added salt e.g. marinades, soy/fish sauce, stock/bouillon |  |  |
|  | added sugar or other sweeteners e.g. honey, syrups |  |  |
| Q5. Does the nutrition content of the product meet any of the following criteria from the Eat for Health Educators Guide? | -- total fat > 10g per 100g | Classify as discretionary or 'mixed product high in fat salt or sugar' | Go to Q6 |
|  | -- saturated fat > 3g per 100g |  |  |
|  | -- total sugar > 15g per 100g |  |  |
|  | -- sodium > 400mg per 100g |  |  |
| Q6. Is there enough information provided to classify the product as five food group foods or mixed product using mainly core foods? | For products where only front-of-pack information is available, products will be classified as discretionary/ mixed product high in fat salt or sugar unless there is sufficient information to classify it as five food group food/ mixed product using mainly five food group foods | Classify into the appropriate food group, or as 'mixed product using mainly five food group foods' | Classify as discretionary or 'mixed product high in fat salt or sugar' |
