# Supplementary figures and images for: Alignment of Supermarket Own Brand Foods’ Front-of-Pack Nutrition Labelling with Measures of Nutritional Quality: An Australian Perspective
Source: Nutrients. 2018 Oct 9;10(10):1465. doi: 10.3390/nu10101465 (PMC6213021; doi:10.3390/nu10101465)

**Supplementary Figure 2: The research process**

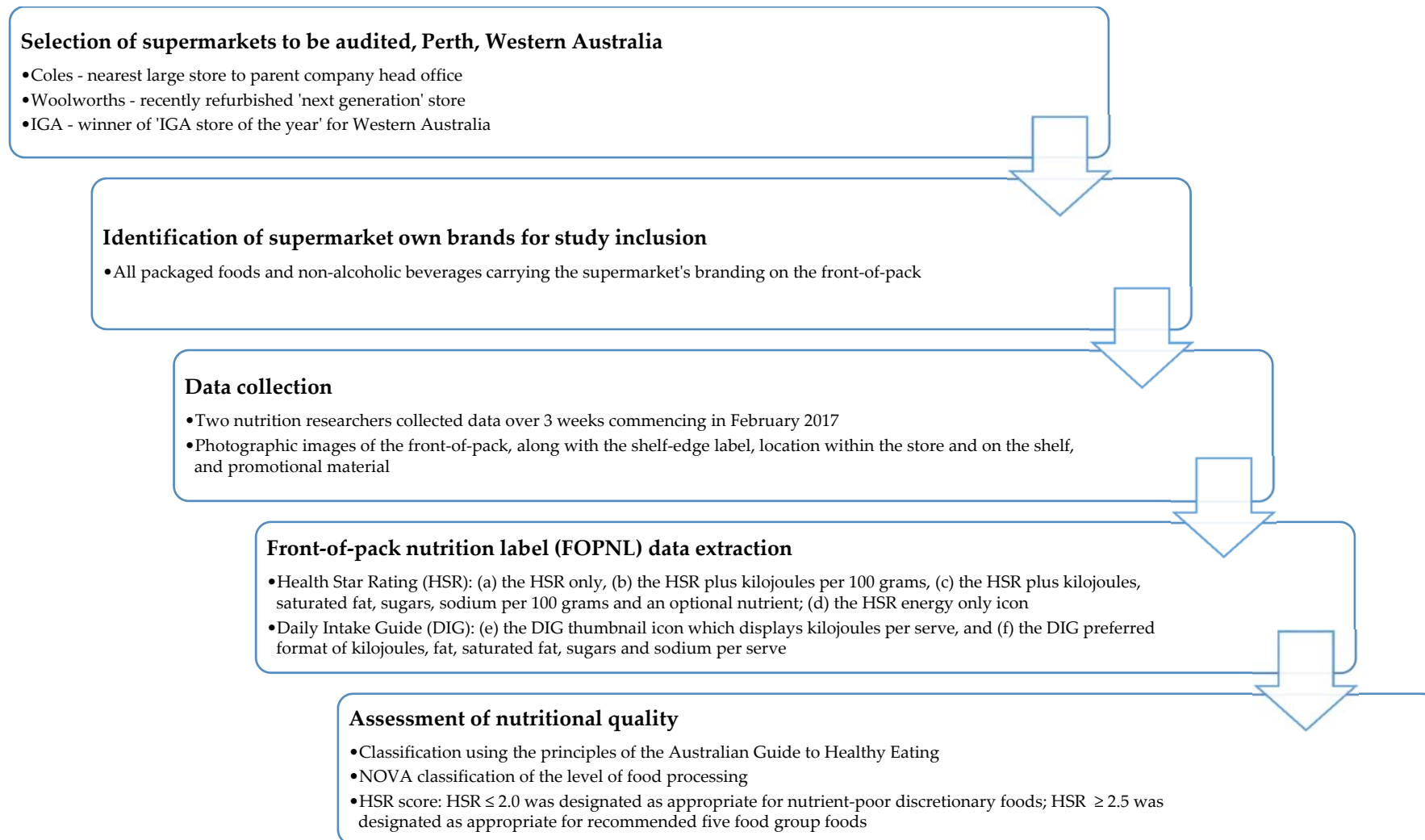

Supplement: Supplementary file 1 [file nutrients-10-01465-s001.zip › suppl/Supplementary Figure 2.pdf]
